# Cannabidiol as a treatment for cocaine use disorder: a scoping review

**DOI:** 10.1007/s00210-026-05037-x

**Published:** 2026-02-03

**Authors:** Leticia Custódio dos Santos, Verônica Barros da Cunha, Jéssyca Milene Ribeiro, Larissa Helena Lobo Torres, Raphael Caio Tamborelli Garcia

**Affiliations:** 1 Department of Pharmaceutical Sciences, Institute of Environmental, Chemical and Pharmaceutical Sciences, Rua São Nicolau, Universidade Federal de São Paulo, São Nicolau, 210, 1° Andar, Diadema, SP 09913-030 Brazil; 2 Department of Food and Drugs, School of Pharmaceutical Sciences, Federal University of Alfenas, Rua Gabriel Monteiro da Silva 700, Alfenas, MG 37130-001 Brazil

**Keywords:** Cannabidiol, Cocaine use disorder, Craving, Pharmacotherapy, Scoping review

## Abstract

Cocaine use disorder (CUD) remains a major public health challenge with limited effective therapeutic options. Cannabidiol (CBD) has been proposed as a potential treatment due to its neuroprotective and anti-craving properties. This scoping review aims to synthesize and map the existing evidence from clinical trials evaluating CBD’s effects on craving reduction, relapse prevention, cognitive function, and emotional regulation in individuals with CUD. Although CBD was well tolerated and associated with fewer adverse events than conventional pharmacological treatments, clinical studies included in this review did not demonstrate significant efficacy over placebo in reducing cocaine craving, preventing relapse, or improving cognitive performance, despite promising preclinical outcomes. The review also highlights methodological limitations across studies, including variability in dosage, treatment duration, and study design. Although current evidence does not support CBD as a standalone treatment for CUD, its favorable safety profile suggests potential utility within a broader, multimodal therapeutic framework. Future research should prioritize standardized protocols and explore synergistic combinations with behavioral or pharmacological interventions to enhance treatment efficacy.

**Supplementary Information:**

The online version contains supplementary material available at 10.1007/s00210-026-05037-x.

## Background

Cocaine use disorder (CUD) and crack use disorder (CrUD) are significant public health concerns, characterized by their high addictive potential, substantial psychosocial impacts, and the limited availability of effective treatment options (Simpson et al. 1997). Globally, the prevalence of cocaine use remains one of the most widely used drugs, with an estimated 23 million users, and the number of crack-cocaine users is continuing to rise and disproportionately affects marginalized populations, overloading healthcare systems and communities according to the World Drug Report (United Nations Office on Drugs and Crime [UNODC], 2024).

Both cocaine and crack cocaine are derived from the coca plant (*Erythroxylum coca*) and share pharmacological properties. However, their chemical compositions and routes of administration differ, leading to variations in their pharmacokinetics and patterns of abuse (Koob and Volkow 2016). Cocaine hydrochloride, commonly known as powdered cocaine, is a water-soluble salt typically snorted or injected intravenously. In contrast, crack cocaine is produced by processing cocaine hydrochloride with a base, such as sodium bicarbonate, resulting in an insoluble, crystalline form that is usually smoked (Garcia et al. 2024).

Traditional treatment options for CUD, such as cognitive-behavioral therapy and pharmacological interventions (e.g., topiramate and bupropion), have shown limited efficacy and are often associated with high relapse rates. Moreover, none of these treatments have received approval from major regulatory agencies worldwide, including the FDA (United States), EMA (Europe), Health Canada, and ANVISA (Brazil), which underscores the urgent need for new therapeutic approaches. Despite scientific advances in understanding the neurobiological underpinnings of these disorders, relapse rates remain alarmingly high, ranging from 40 to 60% within the first year of treatment, according to the National Institute of Drug Abuse.

Over recent years, interest in psychedelic-assisted therapies for substance use disorders (SUD) has grown substantially. Classic psychedelics (e.g., psilocybin) have produced promising signals in early-phase clinical and pilot studies targeting alcohol use disorder and, less convincingly, opioid use disorder. For example, in a double-blind RCT, psilocybin-assisted psychotherapy yielded larger reductions in heavy drinking days relative to an active placebo (Bogenschutz et al. 2022). These interventions are appealing for addiction treatment because they may act via psychological insight, disruption of maladaptive circuits, and promotion of neuroplastic changes (Zafar et al. 2023).

Concurrently, cannabidiol (CBD) is under investigation as a complementary approach: preclinical and limited human studies suggest it may reduce craving and attenuate drug-seeking behavior in opioid, nicotine, and cannabis use disorders (Karimi-Haghighi et al. 2022).

CBD is a non-psychoactive compound derived from *Cannabis sativa* that has emerged as a promising candidate for the treatment of SUD, including CUD. Notably, experimental studies have demonstrated that CBD reduces relapse-like drug-seeking behavior in rodent models with prior cocaine and ethanol self-administration, with these effects persisting beyond the active treatment phase (Gonzalez-Cuevas et al. 2018). In addition, CBD has been shown to counteract psychostimulant-induced neuronal and behavioral sensitization within the mesolimbic dopamine pathway, further supporting its potential utility in targeting the neuroadaptations underlying addictive disorders (Renard et al. 2016).

Clinical trials have explored CBD’s potential in addressing critical challenges associated with CUD, such as intense drug craving, high relapse susceptibility, and cognitive impairments. Craving, a core symptom of addiction, is closely linked to dysregulation of the brain’s reward pathways, particularly in the mesocorticolimbic system (Koob and Volkow 2010). Treatment with CBD has been promising in reducing drug-seeking and relapse in animal models and early-phase human studies (Ren et al. 2009). Additionally, chronic cocaine use is associated with cognitive deficits in executive function, working memory, and attention, further exacerbating the risk of relapse (George et al. 2014).

Emerging evidence indicates that CBD may help mitigate these cannabinoid-induced deficits by promoting neuroprotective mechanisms that preserve dendritic and synaptic structure and by enhancing synaptic plasticity to restore network strength. Renard et al. (2016) demonstrated that chronic adolescent exposure to cannabinoid agonists leads to dendritic atrophy and impaired long-term potentiation in prefrontal circuits, deficits that CBD may help counteract through its neuroprotective and modulatory actions, such as reducing oxidative and inflammatory stress, preserving dendritic architecture via CB1 modulation, and promoting Brain-Derived Neurotrophic Factor-mediated synaptic plasticity (Campos et al. 2012).

Beyond its direct effects on craving and cognition, CBD may also address the emotional and psychological comorbidities frequently observed in individuals with CUD. Anxiety and stress are among the most common triggers for relapse, and CBD’s anxiolytic and stress-buffering properties have been documented in clinical and preclinical studies (Blessing et al. 2015; Zuardi et al. 2017). This multifaceted therapeutic potential of CBD makes it an intervention capable of targeting the addiction.

This scoping review aims to synthesize the evidence from controlled trials (CTs) investigating the therapeutic effects of CBD for CUD. By evaluating outcomes such as craving reduction, relapse prevention, cognitive enhancement, and emotional regulation, it provides a comprehensive understanding of CBD’s potential in treating this disorder, while also highlighting gaps in the current literature and outlining directions for future research to advance the integration of CBD into evidence-based treatment. In accordance with Joanna Briggs Institute (JBI) recommendations, a global organization that supports evidence-based decisions, a comprehensive search was conducted on the Open Science Framework (OSF) platform and in scientific databases, described in the Information Sources section, to ensure that there are no published or registered reviews addressing CBD use in individuals with CUD. This approach aligns with the guidelines provided in the JBI Manual for Evidence Synthesis, which emphasizes the importance of effective search strategies for the overall validity of evidence synthesis.

## Methods

This scoping review was conducted in scientific bases and grey literature using clinical studies. The research question was “Are there sufficient scientific evidence to support the effectiveness of CBD treatment of individuals with CUD?” and the protocol was registered in OSF (10.17605/OSF.IO/V6EM2) to enhance transparency and reproducibility of research.

### Eligibility criteria

Clinical studies comparing cannabidiol versus placebo or other pharmacological treatment in patients with CUD were included. The included studies were conducted with humans of adult age, with no distinction in regard to sex. There were no restrictions on language or publication dates. Concerning the outcome measures, the included data addressed self-reported craving, (e.g., Cocaine Craving Questionnaire–Brief [CCQ-Brief] and Minnesota Cocaine Craving Scale [MCCS]) and/or cue-induced craving assessed via controlled cue-exposure paradigms, as well as cognitive effects, anxiety symptoms, and stress response. Studies evaluating SUDs in general, or those focusing on substances other than crack/cocaine, were excluded. Regarding cannabis treatment, studies with patients exposed to tetrahydrocannabinol (THC) were excluded.

### Information sources

The research was conducted on five databases—PubMed, LILACS, EMBASE, Web of Science, and Scopus (Supplementary material A). The assessment was performed until July 2025 (July 31 st, 2025). A search for gray literature was also conducted on BioRxiv, BDTD, and Google Scholar, but no records were obtained for review.

### Search strategy

A research question was designed according to the PCC (Participant, Concept, and Context) strategy to search in vivo studies—(P): participant, humans exposed to cocaine/crack-cocaine; (C): concept, cocaine/crack-cocaine drug cue craving; and (C): context, exposure to cannabidiol (with intent to treat cocaine/crack-cocaine use). MeSH descriptors were crossed with Boolean operators “and” (inter-category) and “or” (intra-category). No filters were applied during the search in order to maximize the selection of studies for evaluation.

### Selection process

The retrieved studies were imported to Rayyan® for duplicate checking and elimination. Two independent researchers (RCTG and LCS) selected the studies based on the eligibility criteria established for this scoping review. The first screening phase involved reviewing titles and abstracts, followed by a full-text reading of the selected studies. In case of any disagreement, the third reviewer (LHLT) was available to support in order to establish a consensus. The similarity between the studies included and excluded by the three researchers was analyzed using the Kappa coefficient with GraphPad QuickCalcs (GraphPad, San Diego, CA).

### Data collection process

The independent researchers (RCTG and LCS) extracted relevant data from the included studies. The data extraction process provided the following information: (1) general characteristics of the included studies, such as title, author, year, country, study design, experimental groups, duration of exposure, and administration pattern; (2) description of the evaluated outcomes and the results obtained; and (3) main conclusions of the studies. Regular meetings among the reviewers were held to discuss issues, required adjustments, and ensure alignment. A final discussion was conducted to reach consensus on the form and data to be extracted. The information collected by the two reviewers was compared, and no disagreements were observed that required resolution by a third reviewer.

### Study quality and risk of bias assessment

The methodological quality of the clinical studies was assessed using the Downs and Black checklist for clinical trial quality and risk of bias assessment. Although the risk of bias analysis is optional for scoping review articles (Munn et al. 2018), it was conducted to support the evidence regarding the quality of the studies included in the review.

The Downs and Black (1998) checklist is intended to assess the methodological quality of both randomized and nonrandomized comparative studies. It comprises 27 items that cover various methodological aspects, including reporting, external validity, internal validity (bias and confounding), and power of the studies. Reporting questions ensure that the study’s objectives, hypotheses, interventions, and outcomes are clearly detailed. External validity determines if the study’s findings can be generalized to the broader population. Internal validity (bias and confounding) checks if the study has effectively minimized biases and confounding factors that could influence the results. Power (statistical) assesses whether the study has adequate statistical power to detect significant differences between the groups being compared. The items are scored as “yes” (1)—except for item five in the reporting scale, which is scored two (2)—“no” (0), and “unable to determine” (0), providing a quantitative measure of the study’s quality.

## Results

### Study selection

**Fig. 1:**
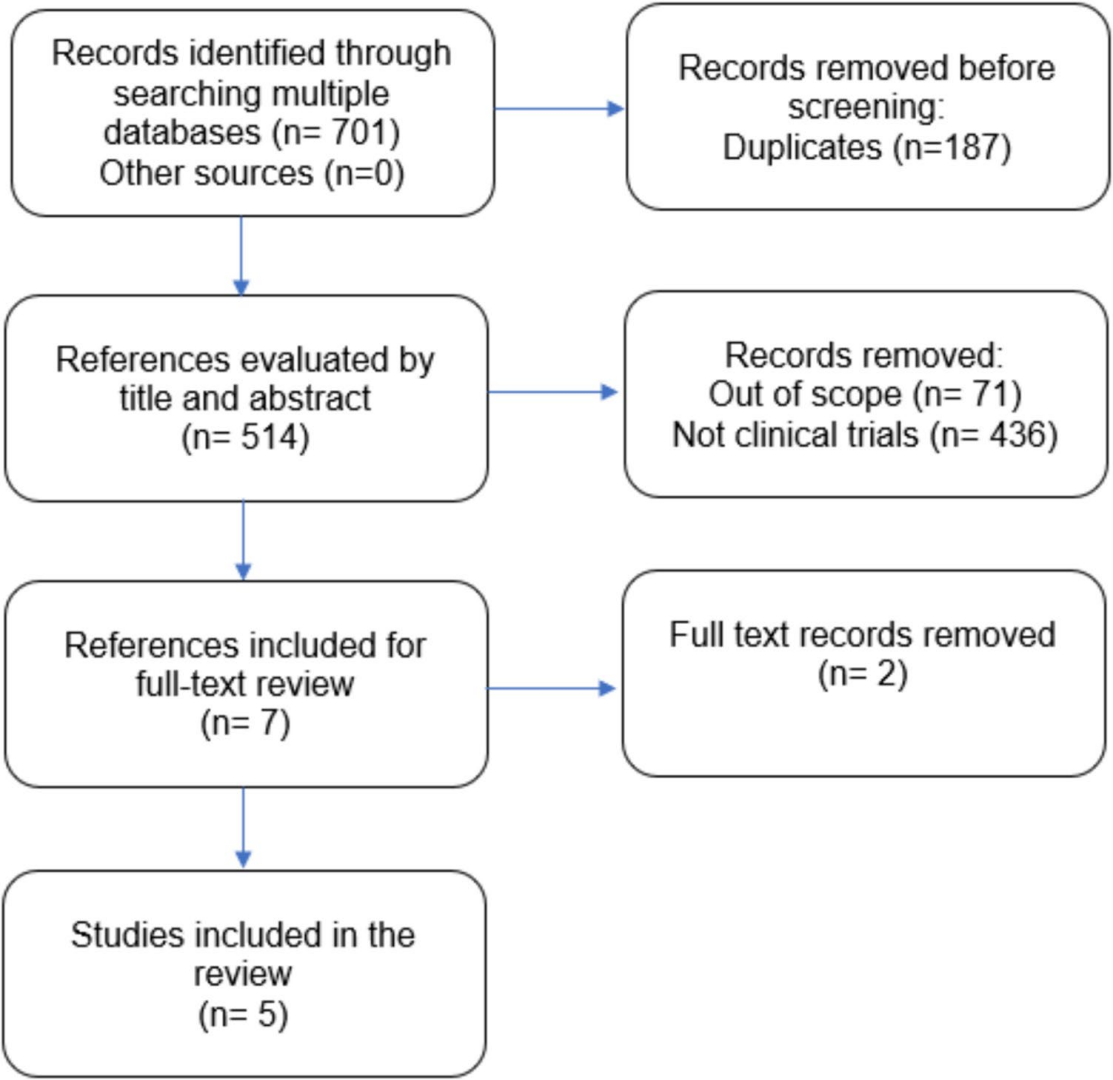
PRISMA flow diagram of the study selection process, adpated from PRISMA-ScR guidelines (Tricco et al. 2018).

The database searches initially yielded 701 records. After removing duplicates, 514 studies remained for further assessment. Screening and application of eligibility criteria resulted in seven studies being selected for full-text review. Following a detailed examination, two studies were excluded (Supplementary Material B). The remaining five studies met the inclusion criteria and were incorporated into this review. No additional studies were identified through manual research. A total of five studies were included in the scoping review, as depicted in the PRISMA flow diagram (Fig. 1). The number of observed agreements among researchers was 100% (kappa = 1.000).

### Study characteristics

The Characteristics of each study are summarized in Table 1. The studies were conducted in Canada and Brazil and published between 2021 and 2024. Among them, three were double-blinded randomized clinical trials. For one of these trials, two exploratory analyses assessing distinct outcomes were also identified. Two studies compared CBD treatment with placebo, whereas the remaining trial compared CBD with a pharmacological treatment. Across studies, synthetic CBD was administered orally in doses ranging from 300 to 800 mg.

**Table 1:** Description of included studies

Author, year, country	Study design	Number of participants/ time of CBD treatment (days)	Objectives	Cocaine/crack use assessment	Main results
Gallassi et al. 2024, Brazil	Double-blind RCT with two treatment groups (CBD and control)	73/ 70 days	Evaluate safety and tolerability, frequency of crack use, adverse events, physical health symptoms, and craving out-of-hospital individuals	Crack use was assessed once per week through a questionnaire and urine samples	Although two significant results in the inter-group analysis were favorable for both groups, CBD (600 mg) group had significant results in more parameters compared to control group in the intra-group analysis [e.g., reducing crack use (*p* = 0.016; 5 weeks)]
Meneses-Gaya et al. 2021, Brazil	Randomized, double-blind, placebo-controlled trial (CBD and placebo)	31/ 10 days	Assess the efficacy of CBD in the management of crack-cocaine craving and the treatment of frequent withdrawal symptoms in hospitalized patients with crack-cocaine dependence	Cocaine Craving Questionnaire– Brief (CCQ-Brief) Minnesota Cocaine Craving Scale (MCCS)	Despite excellent safety and tolerability, 300 mg of CBD failed to demonstrate efficacy in the treatment of craving in subjects with crack-cocaine dependence
Mongeau-Pérusse et al. 2021, Canada	Single-site double-blind randomized controlled superiority trial (CBD and placebo)	78/84 days	Test CBD efficacy for reducing craving and preventing relapse in people with CUD	Urine Timeline follow-back (TLFB) Visual Analogue Scale (VAS) Cocaine Craving Questionnaire– Brief (CCQ-Brief) Cocaine Selective Severity Assessment (CSSA)	800 mg CBD did not reduce cocaine craving or relapse among people being treated for CUD
Mongeau-Pérusse et al. 2022, Canada	Exploratory outcomes from randomized, double-blind, placebo-controlled trial	78/ 84 days (same cohort from Mongeau-Pérusse et al. 2021)	Explore CBD’s effect on stress biomarker (cortisol) and anxiety symptoms in people with CUD	Visual Analogue Scale (VAS) Beck Anxiety Inventory (BAI) Blood	Similar mean BAI anxiety scores in both CBD and placebo groups (estimate (B) = 1.70, Wald = 1.22, *p* = 0.27). No evidence for 800 mg of CBD to be more efficacious than placebo for modulating anxiety symptoms and cortisol levels in individuals with CUD
Rizkallah et al. 2022, Canada	Exploratory outcomes from randomized, double-blind, placebo-controlled trial	78/ 92 days (same cohort from Mongeau-Pérusse et al. 2021)	Explore whether CBD is superior to placebo to improve cognitive functioning in individuals with CUD	Cambridge Neuropsychological Test Automated Battery (CANTAB) Screening Task (MOT) Cambridge Gambling Task (CGT) Pattern Recognition Memory (PRM) Stop Signal Task (SST)	CBD or placebo showed similar quality of decision-making (p = 0.994), deliberation time (p = 0.507), delay aversion (p = 0.968) and risk taking (p = 0.914). No evidence for 800 mg of CBD to be more efficacious than placebo for improving cognitive outcomes

### Synthesis of results and summary of evidence

#### CBD compared to placebo

Applications of synthetic CBD compared to placebo were carried out in order to explore craving during detoxification and time-to-cocaine relapse during subsequent outpatient treatment, effects on cognition, effects on anxiety symptoms, and stress response in individuals with CUD.

For craving scores, Mongeau-Pérusse et al. (2021) observed it was increased from baseline in CBD and placebo groups, and most patients relapsed to cocaine after the study finished. Among all individuals, sixty-two completed the detoxification phase, with an inpatient detoxification phase lasting 10 days, and 50 participants completed the study. The CBD treatment was well tolerated by the participants, and the main adverse event observed was diarrhea. On the other hand, Meneses-Gaya et al. (2021) reported a within-group reduction in craving scores over time in participants receiving CBD; however, these reductions were not significantly different from those observed in the placebo group. For this reason, it was concluded that CBD was unable to interfere with symptoms of crack-cocaine craving.

Regarding effects on improving cognitive functions, according to the study from Rizkallah et al. (2022), there was no statistically significant group difference in terms of stop signal reaction time (*p* = 0.644). Also, both groups performed similar quality of decision-making, deliberation time, delay aversion, and risk-taking. There was no evidence of efficacy in improving cognitive outcomes for CBD when compared to placebo. A total of 71.8% of participants completed the phase I assessment and 78.6% of them completed the phase II trial.

Effects on anxiety symptoms and stress response were assessed using the Beck Anxiety Inventory (BAI), visual analog scale (VAS), and cortisol levels at multiple time points throughout the study were measured by Mongeau-Pérusse et al. (2022). The study found that anxiety scores were similar in both CBD and placebo groups (BAI *p* = 0.27, VAS *p* = 0.18), and there was no statistically significant difference in cortisol levels (*p* = 0.76).

#### CBD compared to pharmacological treatment

Applications of CBD compared to pharmacological treatment (fluoxetine, valproic acid, and clonazepam) were conducted by Gallassi et al. (2024) to assess the feasibility, safety, tolerability, and preliminary efficacy of synthetic CBD compared to standard pharmacological treatment for reducing crack use in individuals with CUD. The study also investigated additional parameters, including adverse events, physical health symptoms, and craving.

Regarding safety and tolerability, for control (pharmacological treatment) and CBD groups the results were considered adequate. Although both groups showed a decrease in crack use, the reduction was not significantly different between CBD and standard pharmacological treatment. Regarding adverse effects, there were differences between both groups, with fewer episodes of diarrhea, constipation, nausea, dizziness, memory impairment, low concentration, tremor, ataxia, and nasal congestion in the CBD group. Inter-group physical health symptoms were more pronounced in the control group with reduction of clinical and psychiatric complaints at the first 2 weeks; however, at the end of the study, the CBD group showed significant improvement in overall health ratings. Regarding crack craving, no difference was detected between these two groups.

Across the studies summarized in Table 1, the treatment period for CBD administration ranged from 10 to 92 days, depending on the study design and objectives. Crack or cocaine use and craving were assessed through a combination of self-report instruments and biological measures. In Gallassi et al. (2024), crack use was evaluated once per week using a questionnaire and urine samples. Meneses-Gaya et al. (2021) applied the Cocaine Craving Questionnaire–Brief (CCQ-Brief) and the Minnesota Cocaine Craving Scale (MCCS). Mongeau-Pérusse et al. (2021) employed the Timeline Follow-Back (TLFB) method, Visual Analogue Scale (VAS), CCQ-Brief, and the Cocaine Selective Severity Assessment (CSSA) based on urine verification. Subsequent studies by Mongeau-Pérusse et al. (2022) and Rizkallah et al. (2022) focused on additional psychological and cognitive outcomes while maintaining the overall study framework.

### Methodological quality of the studies: risk of bias

Table 2 provides an overview of the methodological quality of all the included in vivo studies using Downs and Black (1998) checklist. Supplementary Material C provides a detailed assessment regarding Downs and Black checklist for the studies included in the scoping review.

**Table 2:** Methodological quality overview of in vivo studies assessed using Downs & Black checklist

Author, year, country	Reporting	External validity	Bias	Confounding	Power	Total score
Gallassi et al. 2024, Brazil	10	3	6	6	1	26
Meneses-Gaya et al. 2021, Brazil	10	3	6	6	1	26
Mongeau-Pérusse et al. 2021, Canada	10	3	6	6	1	26
Mongeau-Pérusse et al. 2022, Canada	10	3	6	6	1	26
Rizkallah et al. 2022, Canada	10	3	6	6	1	26

For the five clinical trials retrieved, the total score for the studies was 26 out of 28. The scores fall into the “excellent” category according to the Downs & Black checklist scoring system, in which scoring ranges are classified as excellent (26–28), good (20–25), fair (15–19), and poor (< 14). Based on the scores of studies included in the scoping review, it is concluded that all of them have been rigorously designed and reported with the minimal risk of bias and high validity and the results were reliable.

## Discussion

Across the included studies, CBD did not significantly reduce cocaine or crack-cocaine craving or relapse rates compared to placebo. In drug-cue exposure paradigms, increases in craving were similar between the CBD and placebo groups, and the time-to-relapse was also comparable, with most participants relapsing within the follow-up periods. While these findings suggest limited efficacy of CBD as a monotherapy for CUD, important nuances emerge when considering sample characteristics, treatment regimens, and study design (Gallassi et al. 2024; Meneses-Gaya et al. 2021; Mongeau-Pérusse et al. 2021, 2022; Rizkallah et al. 2022).

Most participants in the included trials had severe CUD, characterized by multiple DSM-5 criteria and substantial medical, psychological, and social impairments (American Psychiatric Association 2013). In contrast, individuals with mild CUD typically present fewer symptoms and less functional disruption, potentially responding differently to pharmacological or behavioral interventions (Grant et al. 2016). Furthermore, participants varied in their levels of crack/cocaine consumption and in the duration of detoxification prior to treatment initiation, which ranged from 0 days (no detoxification) to 10 days. These factors may have influenced treatment outcomes, as heavier and more recent users are likely to require more intensive, multimodal interventions beyond CBD monotherapy. Notably, only in the study by Gallassi et al. (2024) was the sample composed of individuals who reported CUD and were undergoing treatment at a Psycho-Social Care Center (CAPS-AD; a community-based drug treatment service).

The studies administered CBD at varying doses (300–800 mg/day) and durations (10–92 days), which complicates direct comparison of outcomes. Meneses-Gaya et al. (2021), for example, used a lower dose (300 mg/day) for a shorter period (10 days), while Mongeau-Pérusse et al. (2021) applied a higher dose (800 mg/day) for a longer period (92 days), yet neither demonstrated significant effects. Such variability underscores the need to clarify the dose–response relationship and treatment duration required to achieve therapeutic benefit. Moreover, the neurobiology of CUD may involve mechanisms distinct from other SUDs, potentially explaining why CBD has shown promise in heroin (Hurd et al. 2019) and tobacco use disorder (Hindocha et al. 2018), but not in cocaine-related disorders.

Preclinical studies suggest that CBD may reduce drug-seeking behaviors across several SUDs, raising questions about why these effects are not consistently observed in clinical settings for CUD. Hay et al. (2018), for example, highlighted reductions in drug-seeking and consumption in animal and human studies involving nicotine, cannabis, and opioids. However, the absence of efficacy in CUD suggests potential differences in underlying reward mechanisms, neuroadaptations, or treatment responsiveness that warrant further investigation.

The trial by Gallassi et al. (2024) compared CBD with treatment as usual (fluoxetine, valproic acid, clonazepam) for CrUD and found comparable reductions in crack use between groups. Importantly, CBD was better tolerated, with fewer adverse events. In contrast, Mongeau-Pérusse et al. (2022) reported no significant difference between CBD and placebo regarding anxiety, stress reactivity, or cortisol response. Both groups exhibited decreases in anxiety over follow-up, suggesting that CBD was not superior to placebo in modulating these outcomes. Taken together, these results indicate that while CBD demonstrates a favorable safety profile and potential in reducing some complaints associated with CrUD, current evidence does not support superiority over placebo for craving, relapse prevention, or anxiety in CUD.

Interestingly, an excluded Canadian study (Supplementary Material B) reported reductions in inflammatory markers in individuals receiving CBD compared to placebo. Although it did not assess cognitive or clinical outcomes, this finding raises the possibility that CBD could exert alternative benefits in CUD, such as mitigating inflammation and reducing long-term physical harms of chronic cocaine use (Violaine Mongeau-Pérusse et al. 2022). This suggests a potential role for CBD in harm reduction strategies, even if its efficacy in core addiction outcomes remains limited.

Across studies, CBD was consistently well tolerated, with only mild adverse events reported, most frequently diarrhea. This favorable safety profile supports continued research on CBD, particularly as part of combination strategies rather than monotherapy. Potential approaches include integrating CBD with cognitive-behavioral therapy (CBT), contingency management, mindfulness-based interventions, or adjunct pharmacotherapies (e.g., SSRIs, naltrexone, disulfiram). Such multimodal strategies may better address both the neurobiological and psychosocial complexities of CUD (Hay et al. and Bhattacharyya et al. 2018).

The included studies present several limitations that should be considered when interpreting the findings. Most notably, the sample sizes were small, limiting statistical power and applicability. Importantly, there was very little consistency in the analyses performed across these studies, which assessed largely different outcome measures. This heterogeneity represents an additional limitation when attempting to interpret the results collectively. Furthermore, only Gallassi et al. (2024) assessed reduction in cocaine use as an outcome measure. None of the other trials evaluated changes in the frequency or amount of cocaine use during follow-up, which limits the understanding of the potential long-term impact of the interventions on actual drug consumption patterns.

## Conclusions

In conclusion, this scoping review identified only a small number of studies investigating the use of CBD for CUD, which limits the strength of the evidence and underscores the need for further research in this area. Overall, according to the studies included, while CBD was generally well tolerated, current findings do not support its superiority over placebo in reducing cocaine craving, preventing relapse, or improving cognitive, anxiety, or stress outcomes. Some evidence suggests a more favorable tolerability profile compared to traditional pharmacological treatments, as well as potential improvements in clinical and psychiatric complaints, but these findings remain preliminary. Moreover, motivation to change can represent a factor influencing treatment outcomes among individuals with CUD, and insufficient consideration of this variable may partly account for the absence of significant effects observed in the reviewed studies. This aspect should therefore be recognized as an important limitation. Future research involving larger, well-characterized samples, standardized assessment tools, and combined treatment approaches will be crucial to further elucidate the therapeutic potential of CBD for CUD.

## Supplementary Information

Below is the link to the electronic supplementary material.

ESM 1 (DOCX 19.0 KB)

ESM 2 (DOCX 14.0 KB)

ESM 3 (DOCX 47.8 KB)

## Data Availability

All source data for this work (or generated in this study) are available upon reasonable request.
