# Supplementary material for: Cannabidiol as a treatment for cocaine use disorder: a scoping review
Source: Naunyn Schmiedebergs Arch Pharmacol. 2026 Feb 3;399(7):9471–9. doi: 10.1007/s00210-026-05037-x (PMC13152972; doi:10.1007/s00210-026-05037-x)
Supplement: Supplementary file 1 — (DOCX 19.0 KB) [file 210_2026_5037_MOESM1_ESM.docx]

**Supplementary material A - Search strategy**

**PUBMED – July 31^st^, 2025**

| **Search** | **Query** | **Results** |
| --- | --- | --- |
| #1 | Search: **(((substance use disorder) OR ("Substance-Related Disorders"[Mesh])) OR ("Cocaine-Related Disorders"[Mesh])) OR (cocaine use disorder)** | 351,394 |
| #2 | Search: **(("Cocaine"[Mesh]) OR ("Crack Cocaine"[Mesh])) OR ("Cocaine Smoking"[Mesh]** | 27,958 |
| #3 | Search: **"Cannabidiol"[Mesh]** | 4,336 |
| #4 | Search: **((#1) AND (#2)) AND (#3)** | 25 |

**LILACS – July 31^st^, 2025**

| **Search** | **Query** | **Results** |
| --- | --- | --- |
| #1 | **((Substance use disorder)) OR ((substance-related disorders)) OR ((cocaine-related disorders)) OR ((cocaine use disorder))** | 6,166 |
| #2 | **(cocaine) OR ((crack cocaine)) OR ((cocaine smoking) OR (Cocaine Smoking, Crack) OR (Crack Cocaine Smoking) OR (Crack Smoking) OR (Smoking, Cocaine) OR (Smoking, Crack) OR (Smoking, Crack Cocaine))** | 1,822 |
| #3 | **(Cannabidiol)** | 127 |
| #4 | Search: **((#1) AND (#2)) AND (#3)** | 1 |

**EMBASE – July 31^st^, 2025**

| **Search** | **Query** | **Results** |
| --- | --- | --- |
| #1 | **'substance use disorder'/exp OR 'substance use disorder' OR 'substance-related disorders'/exp OR 'substance-related disorders' OR 'cocaine-related disorders'/exp OR 'cocaine-related disorders' OR 'cocaine use disorder'/exp OR 'cocaine use disorder'** | 337,057 |
| #2 | **'cocaine'/exp OR 'cocaine' OR 'crack cocaine'/exp OR 'crack cocaine' OR 'cocaine smoking'/exp OR 'cocaine smoking'** | 83,547 |
| #3 | **'cannabidiol'/exp OR 'cannabidiol'** | 15,129 |
| #4 | Search: **((#1) AND (#2)) AND (#3)** | 141 |

**Web of Science – July 31^st^, 2025**

| **Search** | **Query** | **Results** |
| --- | --- | --- |
| #1 | **TS=((Substance use disorder) OR (substance-related disorders) OR (cocaine-related disorders) OR (cocaine use disorder))** | 85,031 |
| #2 | **TS=((Cocaine) OR (Crack Cocaine) OR (Cocaine Smoking))** | 70,404 |
| #3 | **TS=(Cannabidiol)** | 9,814 |
| #4 | Search: **((#1) AND (#2)) AND (#3)** | 60 |

**Scopus – July 31^st^, 2025**

| **Search** | **Query** | **Results** |
| --- | --- | --- |
| #1 | **TITLE-ABS-KEY ( substance AND use AND disorder ) OR ( substance-related AND disorders ) OR ( cocaine-related AND disorders ) OR ( cocaine AND use AND disorder )** | 317,577 |
| #2 | **TITLE-ABS-KEY ( cocaine ) OR ( crack AND cocaine ) OR ( cocaine AND smoking ) OR ( cocaine AND smoking, AND crack ) OR ( crack AND cocaine AND smoking ) OR ( crack AND smoking ) OR ( smoking, AND cocaine ) OR ( smoking, AND crack ) OR ( smoking, AND crack AND cocaine )** | 126,136 |
| #3 | **TITLE-ABS-KEY ( cannabidiol )** | 12,817 |
| #4 | Search: **((#1) AND (#2)) AND (#3)** | 474 |
