# Supplementary material for: Cannabidiol as a treatment for cocaine use disorder: a scoping review
Source: Naunyn Schmiedebergs Arch Pharmacol. 2026 Feb 3;399(7):9471–9. doi: 10.1007/s00210-026-05037-x (PMC13152972; doi:10.1007/s00210-026-05037-x)
Supplement: Supplementary file 2 — (DOCX 14.0 KB) [file 210_2026_5037_MOESM2_ESM.docx]

**Supplementary material B – Excluded studies**

| **Reference (year)** | **Title** | **Reason for exclusion** |
| --- | --- | --- |
| Violaine Mongeau-Pérusse et al. (2021) | Exploring cannabidiol effects on inflammatory markers in individuals with cocaine use disorder: a randomized controlled trial | Inclusion criteria not met - Focused on inflammatory markers. |
| Sponsored by [University of Sao Paulo General Hospital](https://trial.medpath.com/organization/fe45e9b8b4ad0c2a/university-of-sao-paulo-general-hospital) (2023) | Randomized Double-blind Placebo-controlled Clinical Trial to Evaluate the Safety and Efficacy of the Use of Cannabis Sativa Extract in the Treatment of Cocaine and Crack Addicts | Ongoing phase IV study including THC extract as part of treatment. |
